# Parallel Specification of Visuomotor Feedback Gains during Bimanual Reaching to Independent Goals

**DOI:** 10.1523/ENEURO.0026-17.2017

**Published:** 2017-03-10

**Authors:** Anouk J. de Brouwer, Tayler Jarvis, Jason P. Gallivan, J. Randall Flanagan

**Affiliations:** 1 Centre for Neuroscience Studies, Queen’s University, Kingston, Ontario, Canada; 2 Department of Psychology, Queen’s University, Kingston, Ontario, Canada; 3 Department of Biomedical and Molecular Sciences, Queen’s University, Kingston, Ontario, Canada

**Keywords:** motor control, online corrections, vision, visual perturbations

## Abstract

During goal-directed reaching, rapid visuomotor feedback processes enable the human motor system to quickly correct for errors in the trajectory of the hand that arise from motor noise and, in some cases, external perturbations. To date, these visuomotor responses, the gain of which is sensitive to features of the task and environment, have primarily been examined in the context of unimanual reaching movements toward a single target. However, many natural tasks involve moving both hands together, often to separate targets, such that errors can occur in parallel and at different spatial locations. Here, we examined the resource capacity of automatic visuomotor corrective mechanisms by comparing feedback gains during bimanual reaches, toward two targets, to feedback gains during unimanual reaches toward single targets. To investigate the sensitivity of the feedback gains and their relation to visual-spatial processing, we manipulated the widths of the targets and participants’ gaze location. We found that the gain of corrective responses to cursor displacements, while strongly modulated by target width and gaze position, were only slightly reduced during bimanual control. Our results show that automatic visuomotor corrective mechanisms can efficiently operate in parallel across multiple spatial locations.

## Significance Statement

During goal-directed reaching, rapid visuomotor feedback processes enable the motor system to quickly correct for viewed errors in the trajectory of the hand. To date, these visuomotor responses have mostly been examined in the context of unimanual reaching movements to a single target. However, many natural tasks involve moving both hands at the same time such that errors can occur in parallel and at different locations. We examined the resource capacity of automatic visuomotor corrective mechanisms by comparing feedback gains during bimanual reaches, toward two separate targets, to feedback gains during unimanual reaches toward single targets. We show that automatic visuomotor corrective mechanisms can efficiently operate in parallel across multiple spatial locations, with little cost for bimanual control.

## Introduction

Goal-directed reaching is supported by rapidly elicited motor responses that compensate for viewed errors in hand position, which can arise from both motor noise or external perturbations (Brenner and Smeets, 2003; Saunders and Knill, 2003, 2004; Franklin and Wolpert, 2008; Diamond et al. 2015). These responses have been typically investigated by displacing the position of the cursor controlled by the hand during movement; due to their speed of implementation, they are often referred to as “automatic” responses. A prominent feature of these visuomotor reflexes is that they are flexibly adapted to the task and environment (Franklin and Wolpert, 2008; Franklin et al. 2012). For example, the reflex gain is lower when reaching toward a wide, compared with a narrow, target (Knill et al. 2011; Gallivan et al. 2016), consistent with the policy of minimum intervention whereby the sensorimotor system responds more robustly to errors that endanger the goal of the task compared with those that do not (Todorov and Jordan, 2002; Scott, 2004).

To date, rapid visuomotor responses have mostly been examined in the context of unimanual reaches to a single target. However, many of the natural action tasks we perform on a daily basis involve bimanual control, wherein the two hands are simultaneously directed toward different spatial goals and errors can thus occur on either hand and hence at different spatial locations. Although previous work has examined responses to cursor displacements during bimanual movements (Reichenbach et al. 2013), it is not known whether these responses exhibit a limited resource capacity and are diminished in comparison to unimanual movements. To date, the resource capacity of visual-spatial processing has predominantly been investigated using perceptual tasks. Whereas visual attention is classically thought of as being allocated to one location in the visual field at a time (e.g., Posner, 1980), other work has suggested that individuals can concurrently attend to multiple visual locations, with minimal performance cost (Pylyshyn and Storm, 1988; Awh and Pashler, 2000; Müller et al. 2003; Alvarez and Cavanagh, 2005). In the context of action-related processing, the capacity to generate corrective responses in a bimanual reaching task in which the two hands simultaneously reached to separate targets has been examined for target displacements (Diedrichsen et al. 2004). This study found that each hand’s response to a displacement of its corresponding target was equally efficient in bimanual and unimanual reaching, suggesting that these target-related corrective responses operate largely in parallel. However, because corrections to target and hand cursor displacements appear to involve distinct mechanisms (Reichenbach et al. 2014; Franklin et al. 2016), the parallel processing capacity of the latter remains to be determined.

Here, using a planar robotic interface and virtual reality system, we examine the resource capacity of automatic visuomotor corrective mechanisms by comparing corrections in response to cursor displacements during bimanual reaching to two targets and unimanual reaching to a single target. Because the ability to detect viewed errors in hand position may depend on the direction of movement relative to visual fixation (Paillard, 1982) and eccentricity in peripheral vision, we manipulated gaze location by instructing participants to fixate the left-hand target, the right-hand target, or a central position. We show that the feedback gain of the corrective response, while strongly modulated by gaze position, exhibits a reliable but small cost for bimanual control, indicating that automatic visuomotor corrective mechanisms can operate efficiently and in parallel across multiple spatial locations.

## Materials and Methods

### Participants

Fifteen people participated in experiment 1 (ages 19–33 years, 7 men), and 15 different people participated in experiment 2 (ages 19–26 years, 5 men). The data of one participant in experiment 1 was discarded because of technical problems, and the data of one participant in experiment 2 was excluded from analysis because the gaze data following the cursor perturbation was missing in more than half of the trials. All participants self-reported being right-handed and had normal or corrected-to-normal vision. Participants were compensated for their time with a cash payment of $60 for experiment 1 or $25 for experiment 2. The study was approved by the Queen’s University Research Ethics Board, and participants provided written informed consent before participating.

### Experimental Setup

**Figure 1.**
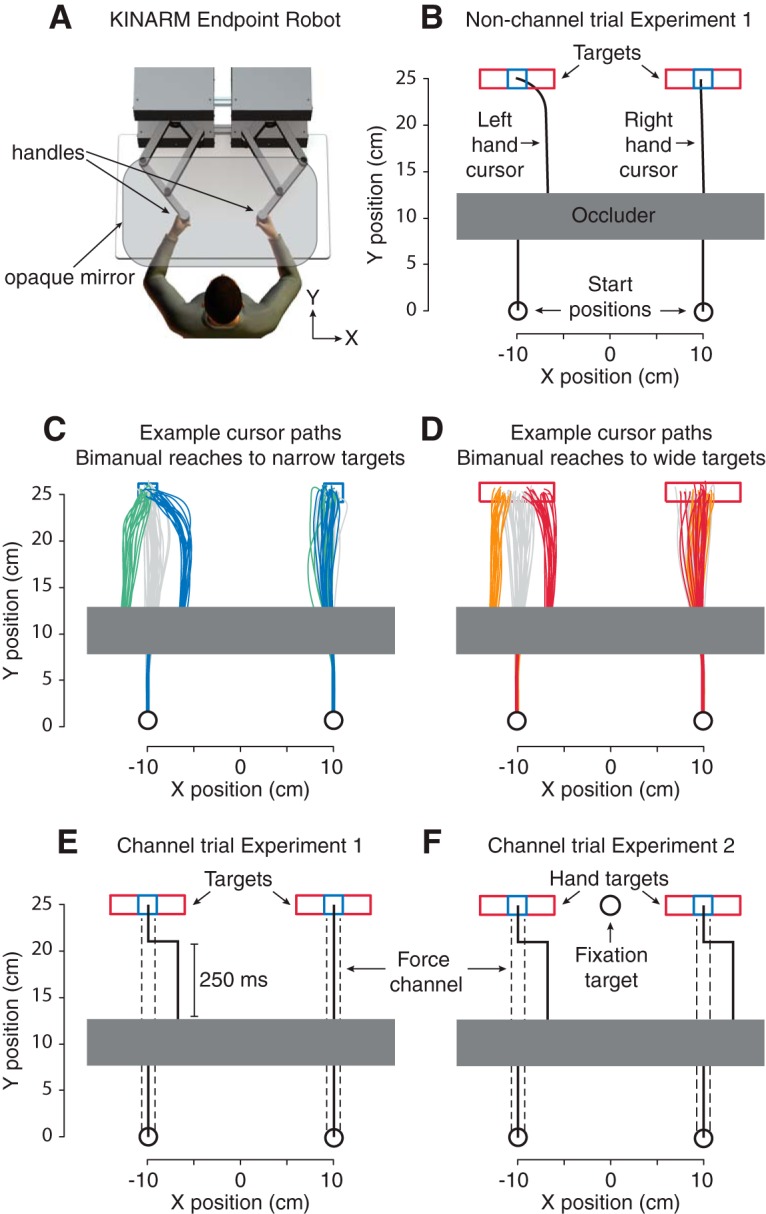
Experimental methods. (A) Experimental setup. Participants performed reaching movements in the horizontal plane while holding on to the handles of the robotic manipulandum. (B) Example bimanual nonchannel trial of experiment 1. Participants were instructed to fixate on the left or right reach target. Reach targets could be both narrow (in blue) or wide (in red). On a subset of trials, one of the hand cursors was visually displaced to the left or right after it passed under a visual occluder, requiring a correction of the movement trajectory. (C) Cursor paths from an example participant in response to a leftward (in green), zero (in gray), and rightward (in blue) shift of the left-hand cursor during bimanual reaching to narrow targets in nonchannel trials. (D) Same as C, but with reaching to wide targets (orange, leftward cursor shift; gray, no cursor shift; red, rightward cursor shift). (E) Example bimanual force channel trial of experiment 1. Participants’ hand movements were constrained along a straight line from start to target position, allowing us to measure the forces applied into the virtual wall of the channel (depicted by the black dashed lines). In cursor perturbation trials, the cursor automatically moved back to this line 250 ms after the perturbation. (F) Example bimanual force channel trial of experiment 2. Participants were instructed to fixate on a central fixation target. On a subset of trials, a single or both hand cursors were visually displaced to the left or right.

Participants were seated in a chair with the forehead resting against a pad and their hands holding onto the handles of a robotic manipulandum (KINARM End-Point Robot, BKIN Technologies; Fig. 1*A*). They performed unimanual and bimanual target-directed reaches by moving the handles away from the body in the horizontal plane. Kinematics and forces at the handles were measured at 1129 Hz. Eye movements were recorded using a built-in video-based eye tracker (Eyelink 1000; SR Research) at 500 Hz. Stimuli were projected onto an opaque mirror positioned horizontally between a monitor and the handles, such that the stimuli appeared in the plane of the handles. The mirror prevented vision of the participants’ arms.

### Stimuli

The hand positions were represented as two cursors (1-cm-diameter circles) that were aligned with the handles. Movements were made from two starting positions (2-cm-diameter circles) to two narrow or wide rectangular targets (narrow, 2 × 2 cm; wide, 8 × 2 cm) located 10 cm to the left and right of the midline (Fig. 1*B*). The centers of the targets were located 25 cm in front of the starting positions. A 50 × 5-cm visual occluder, under which the hand cursors would pass, was located in between the starting positions and targets such that the far edge of the occluder was the halfway distance of the reaching movement (i.e., 12.5 cm). In unimanual trials (experiment 1), the reach target was presented as a filled square/rectangle (depending on target size), and the other target was presented as an outlined square/rectangle. In bimanual trials (experiments 1 and 2) both reach targets were filled. Participants were instructed to fixate on one of the targets (experiment 1) or a fixation target, positioned in between the two targets (2-cm-diameter circle, experiment 2; Fig. 1*F*) during the reach movement.

### Procedure

Each trial began with participants moving the two cursors into the two starting positions and keeping this position for 250 ms. Next, the targets and occluder were presented until the end of the trial. The target to be fixated was briefly flashed five times with an interval of 100 ms, indicating to the participant to direct their gaze to this target. Participants were instructed to maintain fixation until they completed the reach movement. At 750 ms after flashing the fixation target, five successive beeps (tone frequency 400 Hz; duration 80 ms) started playing, 600 ms apart, cueing participants to first prepare (beeps 1–3) and then execute (beeps 4 and 5) the reach movement. Specifically, participants were instructed to initiate their movement on the fourth beep and arrive at the target on the fifth beep. On all trials, the cursors passed beneath the visual occluder. On cursor perturbation trials, the cursors were displaced 3 cm to the left or right of the handle position underneath the occluder such that, when it reappeared at the far edge of the occluder, participants would correct its position to hit the target (see Fig. 1*C*, *D* for hand paths from an example participant). The first 7.5 cm of the movement was constrained by a mechanical channel (stiffness 6000 N/m, damping 1.5 N/m/s) generated by the KINARM, after which the channel was ramped down in 50 ms, to ensure that the cursors exited the occluder close to the line between the start position and the center of the target or, in perturbation trials, 3 cm to the left or right of this line. The trial ended when the hands reached the target. After trial completion, a text message, displayed centrally on the screen, provided feedback on movement time (either “good,” “too fast,” or “too slow”). In experiment 1, an error in the feedback calculation caused movement times to be slightly longer than the targeted movement time of 600 ms. In experiment 2, the total movement time (i.e., from the hand leaving the start position to reaching the target) was considered good if it was between 500 and 900 ms.

#### Channel trials

We used channel trials to assess the gain of the corrective responses. In these trials, the movement of the participants’ hands was restricted along a straight-line path from the start to target position by a mechanical channel (stiffness 6000 N/m, damping 1.5 N/m/s; Fig. 1*E*, *F*). This allowed us to measure the corrective forces exerted into the channel wall in response to the visual perturbation. The use of channel trials is considered a highly sensitive and reliable method for measuring corrective responses in a manner that is uncontaminated by limb dynamics (Scheidt et al. 2000; Franklin and Wolpert, 2008). In channel trials with a cursor perturbation, the cursor was automatically shifted back to a position on a straight line connecting the start position and the target 250 ms after the perturbation, consistent with previous work (Dimitriou et al. 2013; Gallivan et al. 2016). Because this shift occurred around the time of the correction, participants generally believed that they were responsible for bringing the cursor back in-line to the target. To further prevent an adaptive decrease in the magnitude of the corrective response across trials (Franklin and Wolpert, 2008), only half of the trials of each experiment consisted of channel trials. Channel trials and nonchannel trials were randomly interspersed.

#### Experiment 1

In our first experiment, we investigated the capacity of the visuomotor system to respond to visual errors when two hands are moving compared with when only one hand is moving, and how these responses are modulated by gaze position. To this end, participants performed reaching movements in four conditions: using a single or both hands and fixating gaze on the target at the same side as the cursor perturbation or the target opposite to the side of the cursor perturbation. We had 32 trial types: 2 reach modes (unimanual/bimanual) × 2 fixation sides (left/right) × 2 target sizes (narrow/wide) × 2 perturbation sides (left/right hand cursor) × 2 cursor perturbation directions (leftward/rightward). Each participant performed 32 repetitions per trial type. In addition, each participant performed 16 repetitions × 8 (left/right hand reach × left/right fixation × narrow/wide targets) unimanual trials without a cursor perturbation, and 32 repetitions × 4 (left/right fixation × narrow/wide targets) bimanual trials without a cursor perturbation, altogether resulting in a total of 1280 trials. As noted above, half of these trials were channel trials.

Participants performed two testing sessions on separate days (mean ± SEM 8 ± 2 days apart), consisting of 1 practice block of 44 (all nonchannel trial types; session 1) or 20 (random sampling of nonchannel trial types; session 2) trials and then 4 experimental blocks of 160 trials (∼25 min per block). Trials were randomly intermixed within each block.

#### Experiment 2

In our second experiment, we investigated whether the visuomotor system can set different, independent feedback gains for the two arms during bimanual reaching. Participants performed bimanual reaching movements to two targets while fixating gaze on a central fixation target (see Fig. 1*F*). We chose to include a central fixation point so as not to bias the processing of visual information at one hand versus the other, while also maximizing the opportunity that the visual system capitalizes on its independent resource capacity for the two hemifields, as observed in perceptual tracking of multiple targets (Alvarez and Cavanagh, 2005).

Participants were presented with three different perturbation conditions: perturbation of the left or right hand cursor (single perturbation trials), perturbation of both cursors in the same direction (double-same perturbation trials), and perturbation of both cursors in opposite directions (double-opposite perturbation trials). In addition, there were four target width combinations: two narrow targets, two wide targets, and one narrow and one wide target (i.e., left target narrow and right target wide or vice versa). There were 16 trial types in the single perturbation condition: 4 target width combinations × 2 perturbation sides (left/right hand cursor) × 2 perturbation directions (leftward/rightward). The double-same perturbation condition consisted of 8 trial types: 4 target width combinations × 2 perturbation directions (leftward/rightward). The double-opposite perturbation condition consisted of 8 trial types: 4 target width combinations × 2 perturbation directions (inward/outward). Each participant performed 16 repetitions of each trial type, plus 32 × 4 (target width combinations) unperturbed trials, resulting in a total of 640 trials. Half of these trials were channel trials.

Participants performed two testing sessions on separate days (mean ± SEM 6 ± 1 days apart), consisting of 1 practice block of 36 (all nonchannel trial types; session 1) or 20 (random sampling of nonchannel trial types; session 2) trials and then 4 experimental blocks of 80 trials (∼10 min per block). Trials were randomly intermixed within each block.

### Data Analysis

Data were analyzed using Matlab R2015b. Statistical tests were performed using SPSS 23 using an α level of 0.05, adjusted using Bonferroni correction where appropriate (Table 1).

**Table 1.** Statistical analysis

Experiment	Variable	Statistical test	Factor or comparison	Test values
1	Corrective force differences	2 × 2×2 repeated-measures ANOVA	TWFSHTW×FIXTW×HFIX×H	*F*_(1,13)_ = 173.0, *p* < 0.001, OP = 1.0*F*_(1,13)_ = 59.0, *p* < 0.001, OP = 1.0*F*_(1,13)_ = 17.6, *p* = 0.001, OP = 0.97*F*_(1,13)_ = 33.4, *p* < 0.001, OP = 1.0*F*_(1,13)_ = 1.5, *p* = 0.238, OP = 0.21*F*_(1,13)_ = 1.4, *p* = 0.262, OP = 0.19
1	Ratio of corrective force differences	2 × 2 repeated-measures ANOVA	FIXHFIX×H	*F*_(1,13)_ < 0.1, *p* = 0.976, OP = 0.05*F*_(1,13)_ = 0.1, *p* = 0.746, OP = 0.06*F*_(1,13)_ = 0.27, *p* = 0.611, OP = 0.08
1	Corrective force differences at nonperturbed hand	One-sample *t*-tests (corrected α = 0.0125)	nt+fixnt+nfixwt+fixwt+nfix	*t*_(13)_ = 4.2, *p* = 0.001, CI [0.06–0.19]*t*_(13)_ = 3.7, *p* = 0.003, CI [0.04–0.15]*t*_(13)_ = 2.2, *p* = 0.050, CI [<0.0001–0.15]*t*_(13)_ = 1.5, *p* = 0.157, CI [–0.02 to 0.11]
1	Correction onsets (*t*-test method)	2 × 2 repeated-measures ANOVA	FIXHFIX×H	*F*_(1,13)_ = 120.4, *p* < 0.001, OP = 1.0*F*_(1,13)_ = 2.1, *p* = 0.166, OP = 0.27*F*_(1,13)_ = 0.23, *p* = 0.643, OP = 0.07
1	Correction onsets (extrapolation method)	2 × 2 repeated-measures ANOVA	FIXHFIX×H	*F*_(1,13)_ = 70.9, *p* < 0.001, OP = 1.0*F*_(1,13)_ = 2.9, *p* = 0.113, OP = 0.35*F*_(1,13)_ = 1.0, *p* = 0.344, OP = 0.15
1	Gaze position	2 × 2×2 repeated-measures ANOVA	TWFSHTW×FIXTW×HFIX×H	*F*_(1,13)_ = 14.8, *p* = 0.002, OP = 0.94*F*_(1,13)_ = 4.0, *p* = 0.067, OP = 0.46*F*_(1,13)_ = 43.9, *p* < 0.001, OP = 1.0*F*_(1,13)_ = 16.1, *p* = 0.001, OP = 0.96*F*_(1,13)_ = 17.2, *p* = 0.001, OP = 0.97F_(1,13)_ = 0.09, *p* = 0.766, OP = 0.06
2	Corrective force differences	2 × 2×2 repeated-measures ANOVA	TWTW-OPCTW×TW-OTW×PCTW-O×PC	*F*_(1,13)_ = 99.9, *p* < 0.001, OP = 1.0*F*_(1,13)_ = 10.4, *p* = 0.007, OP = 0.85*F*_(2,26)_ = 9.7, *p* = 0.001, OP = 0.97*F*_(1,13)_ = 19.6, *p* = 0.001, OP = 0.98*F*_(2,26)_ = 1.9, *p* = 0.170, OP = 0.36*F*_(2,26)_ = 0.5, *p* = 0.620, OP = 0.12
2	Corrective force differences	Planned comparisons	nt(nt) vs. nt(wt)wt(nt) vs. wt(wt)	*p* = 0.211, OP = 0.23*p* < 0.001, OP = 0.99
2	Corrective force differences	Pairwise comparisons	sp vs. dp-ssp vs. dp-odp-s vs. dp-o	*p* = 0.001, CI [0.06–0.18]*p* = 0.161, CI [–0.03 to 0.18]*p* = 0.002, CI [0.08–0.31]
2	Correction onsets (*t*-test method)	One-way ANOVA	PC	*F*_(2,26)_ = 0.8, *p* = 0.441, OP = 0.18
2	Correction onsets (extrapolation method)	One-way ANOVA	PC	*F*_(2,26)_ = 0.2, *p* = 0.856, OP = 0.072
2	Corrective force differences at nonperturbed hand	One-sample *t*-tests (corrected α = 0.0125)	nt(nt)+spnt(wt)+spwt(nt)+spwt(wt)+sp	*t*_(13)_ = –0.29, *p* = 0.775, CI [–0.10 to 0.08]*t*_(13)_ = –0.30, *p* = 0.767, CI [–0.14 to 0.10]*t*_(13)_ = –0.23, *p* = 0.821, CI [–0.13 to 0.10]*t*_(13)_ = 2.2, *p* = 0.051, CI [–0.0002 to 0.15]

OP, observed power; CI, 95% confidence interval; TW, target width; TW-O, target width, other hand; FS , fixation side; H, hands; PC, perturbation condition; nt/wt, narrow/wide target; fix/nfix, fixation/nonfixation side; sp/dp-s/dp-o, single/double-same/double-opposite perturbation.

#### Channel trials

Kinematic and force data were resampled to 1000 Hz. The forces measured in the channel of the left and right hand were aligned to the perturbation of the left and right cursor, respectively, that is, the moment that the cursor reappeared at the far edge of the occluder. Trials were excluded from the analyses if the time difference between the left- and right-hand cursor reappearing from the occluder was larger than 100 ms or if the movement time of either hand was longer than 1200 ms (experiment 1) or 1000 ms (experiment 2). Movement time was defined for each hand separately as the time difference between movement onset (i.e., the moment when the cursor had fully moved out of the starting position) and the moment the target was reached (i.e., the moment where the center of the cursor was inside the rectangular target area). The average movement time was 817 ± 30 ms in experiment 1 and 499 ± 16 ms in experiment 2.

To obtain a measure of the strength of the automatic visuomotor correction (i.e., feedback gain), forces were first averaged across an interval from 180 to 230 ms after cursor perturbation (Franklin and Wolpert, 2008). Trials were excluded from the analyses if the average force in this window was outside a range of the mean force ±3 standard deviations for each participant and trial type. The mean of the corrective forces following a rightward cursor perturbation was subtracted from the mean of the corrective forces following a leftward cursor perturbation, so that a correct response results in a positive difference value. The resulting corrective force differences were averaged across the left and right hand.

We also computed, using bimanual trials with a single cursor perturbation, a measure of crosstalk between the two hands. The strength of crosstalk was computed by averaging forces at the nonperturbed hand across an interval from 180 to 230 ms after perturbation onset and performing the same subtraction as for the forces at the perturbed hand. A positive value indicates that there is crosstalk between the hands whereby the nonperturbed hand responds in the same direction as the required response at the perturbed hand. For example, if the left cursor is shifted leftwards, such that the correct response of the left hand would involve a rightward force, crosstalk would be manifest as a rightward force at the right hand.

To compute the onset times of the force corrections, we compared the individual force profiles following leftward and rightward perturbations of a single hand and trial type. First, unpaired *t*-tests were applied to each time point to find the minimum *p*-value. Next, searching back from the minimum *p*-value, the onset of the correction was defined as the first sample for which *p* < 0.05. These values were separately verified by using an extrapolation method applied to the averaged difference between force profiles for leftward and rightward perturbations per participant and trial type. To determine the onset times, we fitted a line through the points at which the average force difference reached 25% and 50% of the first peak difference in force response and determined at which time point this line crossed zero (Oostwoud Wijdenes et al. 2014). Onset times were computed only for trial types with narrow targets, because the computation of onset times for wide targets yielded unreliable results because of the lower force responses. Force correction onsets were averaged across hands.

#### Gaze data

Blinks and missing samples from the eye tracker were interpolated where possible. We computed the average gaze position during the first 20 ms after the perturbation. For bimanual movements, we used the average time point of the two hand cursors reappearing at the far edge of the occluder. To constrain our analyses, we examined only the gaze data in channel trials. Trials were excluded from the analyses if the horizontal gaze position was incorrect. Specifically, in experiment 1, we considered gaze position incorrect if its horizontal distance to the center of the target was larger than 10 cm (i.e., gaze went across the midline, in between the targets). In experiment 2, we considered gaze position incorrect if its distance to the center of the fixation dot was larger than 5 cm (i.e., gaze went across the midline in between the fixation dot and the center of the left or right target). Trials with errors in the *y*-direction were not removed from analysis because these were typically due to problems with eye tracking in the horizontal plane (e.g., cases in which the eyelids partly occluded the eyes while participants were looking down at the mirror).

## Results

### Experiment 1

**Figure 2.**
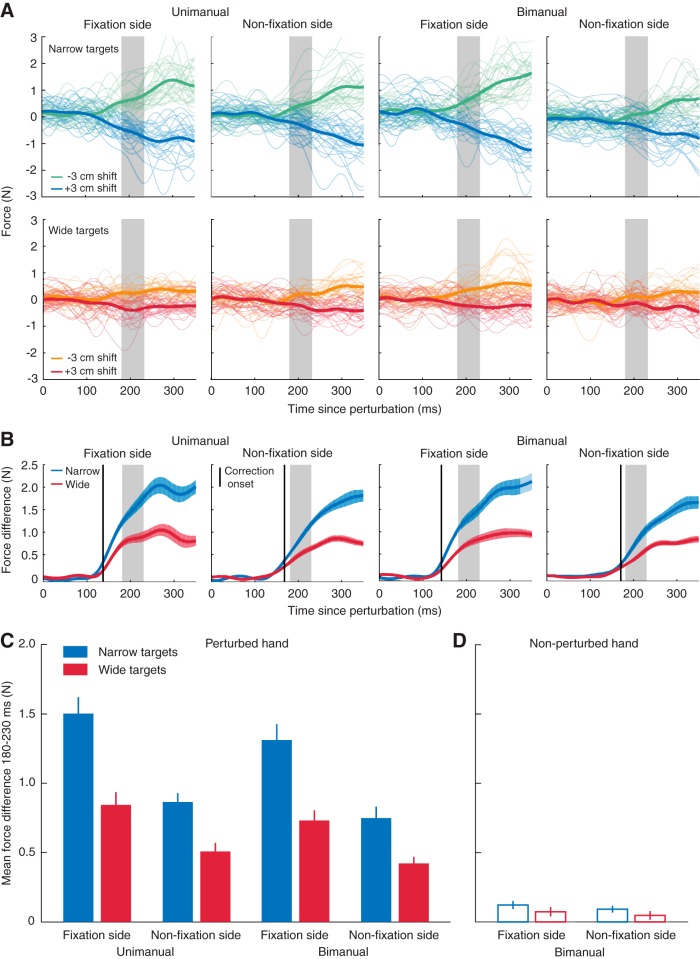
Visuomotor responses in experiment 1. (A) Raw forces measured in channel trials in response to a leftward (–3 cm; in green and orange) and rightward (3 cm; in blue and red) displacement of the visual cursor during reaches to narrow (top row) and wide targets (bottom row) of the same example participant as in Fig. 1. (B) Difference in force responses to leftward and rightward cursor perturbations during reaches to narrow (in blue) and wide targets (in red), averaged across participants. Blue and red shaded areas indicate ±1 SEM. The black vertical line indicates the average onset of the corrective response (see Methods). The gray shaded area indicates the 180- to 230-ms interval across which the force differences were averaged to obtain a single measure of the strength of the response. (C) Mean force differences averaged across the 180- to 230-ms interval following the cursor perturbation. Error bars represent ±1 SEM. (D) Mean force differences at the nonperturbed hand in bimanual conditions.

In our first study, we compared visuomotor feedback gains during bimanual versus unimanual control, and examined how these gains depend on gaze location. Specifically, we measured corrections in response to lateral displacements of the hand cursor during unimanual reaches and one of the two hand cursors during bimanual reaches. Gaze was directed to either the left- or right-hand target, both of which were visible in all trials, and these targets were either both wide or both narrow. A shift in cursor position halfway through the reach elicited rapid corrections of the movement trajectory (see Fig. 1*C*, *D* showing cursor paths from an example participant) which, in channel trials, resulted in a rapid change in force exerted against the wall of the force channel. Fig. 2*A* shows the raw (thin lines) and mean force traces (thick lines) of a representative participant in response to leftward and rightward cursor displacements in each experimental condition. Fig. 2*B* shows the force trajectories for rightward cursor shifts subtracted from the force trajectories for leftward cursor shifts, averaged across participants. To obtain a single, direction-invariant measure of the strength of the corrective response for each experimental condition, we computed the average force response across the 180- to 230-ms interval after the cursor shift (i.e., 25–75 ms after correction onset) and subtracted the mean force after a leftward cursor shift from the mean force following a rightward cursor shift (Fig. 2*C*). Repeated-measures ANOVA performed on these values showed that corrective forces were significantly influenced by target size (*F*_(1,13)_ = 173.0, *p* < 0.001), fixation position (*F*_(1,13)_ = 59.0, *p* < 0.001), and whether the movement was performed with one or two hands (*F*_(1,13)_ = 17.6, *p =* 0.001). This shows that corrections were (1) larger for narrow than wide targets, (2) larger for perturbations that occurred on the side of space that the target was fixated versus not fixated, and (3) smaller during bimanual than unimanual reaching. In addition, there was a significant interaction between fixation side and target width (*F*_(1,13)_ = 33.4, *p* < 0.001), such that the effect of target width was greater for cursor perturbations at the fixation side than for perturbations at the nonfixation side. However, the sensitivity to target width, computed as the ratio between the corrective force for the narrow versus wide target, was not affected by fixation side (mean ± SEM 1.8 ± 0.07, *F*_(1,13)_ < 0.1, *p =* 0.976).

Next, we tested for crosstalk in the corrective responses in bimanual trials by computing the difference in force at the nonperturbed hand between trials with leftward and rightward cursor shifts of the perturbed hand (Fig. 2*D*) and subjecting these differences to one-sample *t*-tests (Bonferroni adjusted α = 0.0125). We found significant crosstalk, reflected by nonzero force differences, when the targets were narrow (mean ± SEM force for perturbation at fixation side, 0.12 ± 0.03 N, *t*_(13)_ = 4.189, *p =* 0.001; perturbation at nonfixation side, 0.09 ± 0.03 N, *t*_(13)_ = 3.675, *p =* 0.003), but no significant crosstalk when the targets were wide (perturbation at fixation side, 0.07 ± 0.03 N, *t*_(13)_ = 2.169, *p =* 0.050; perturbation at nonfixation side, 0.05 ± 0.03 N, *t*_(13)_ = 1.502, *p =* 0.517). As such, although the strength of responses at the non-perturbed hand was expectedly much less, the pattern of crosstalk on that hand was similar to the pattern of corrective responses observed at the perturbed hand (compare Fig. 2*C* to Fig. 2*D*).

To examine whether the timing of the corrections was modulated by gaze position or reaching with one or two hands, we computed the onset of the correction in each condition by performing *t*-tests between individual force traces for leftward and rightward perturbations of reaches to narrow targets (see Methods). Repeated-measures ANOVA revealed that corrections occurred earlier for perturbations of the cursor moving toward the fixated target than for perturbations of the cursor moving toward the nonfixated target (mean ± SEM 140 ± 2 and 169 ± 4 ms, respectively, *F*_(1,13)_ = 120.4, *p* < 0.001). Notably, correction onsets were not influenced by whether the movement was performed with one or two hands (*F*_(1,13)_ = 2.1, *p =* 0.166) or an interaction between these two factors (*F*_(1,13)_ = 0.2, *p =* 0.643). The extrapolation method applied to the averaged force differences of each participant (see Methods) yielded slightly earlier correction onsets (perturbation at fixation side, 126 ± 3 ms; perturbation at nonfixation side, 146 ± 4 ms), but a very similar pattern of results (effect of fixation side, *F*_(1,13)_ = 70.9, *p* < 0.001; effect of hands, *F*_(1,13)_ = 2.9, *p =* 0.113; interaction, *F*_(1,13)_ = 1.0, *p =* 0.344).

As described above, we opted to use a fixed interval (180–230 ms) over which to average forces rather than adapt the interval to the timing of correction onsets to calculate the strength of corrections. However, for completeness, we also evaluated corrective force differences using the latter approach. Specifically, we adjusted the intervals to the correction onsets, averaging the forces over an interval from 25 to 75 ms after correction onset: from 165 to 215 ms for the conditions with a perturbation at the fixation side, and from 194 to 244 ms for the conditions with a perturbation at the nonfixation side. Importantly, none of the statistical results were affected by this alternate method.

Finally, we examined participants’ horizontal gaze positions at the moment of perturbation. Although participants were required to fixate on the left or right target, we observed small differences in fixation position within the margins of the targets. To quantify these effects, gaze positions in correct trials were computed with respect to the center of the target and mirrored for the right target so that positive values reflect a deviation of gaze toward the vertical midline of the screen. ANOVA revealed that gaze positions deviated more toward the midline for wide than for narrow targets (9 ± 2 vs. 3 ± 1 mm), and for bimanual compared with unimanual reaches (8 ± 2 vs. 4 ± 1 mm), as reflected by a main effect of target width (*F*_(1,13)_ = 14.8, *p =* 0.002), a main effect of whether one or two hands were reaching (*F*_(1,13)_ = 43.9, *p* < 0.001), and an interaction between these two factors (*F*_(1,13)_ = 17.2, *p =* 0.001). We also observed a significant interaction between fixation side and target width (*F*_(1,13)_ = 16.1, *p =* 0.001), indicating that the effect of target width was more pronounced for fixations at the right than left target.

In summary, we found that visuomotor corrections to lateral displacements in cursor position were larger when reaching to narrow than to wide targets, and that corrections were both faster and larger for perturbations of the cursor moving toward the fixated versus nonfixated target. Notably, although corrections during bimanual reaching were 13% weaker than during unimanual reaching, we observed no difference in the timing of the corrections.

### Experiment 2

**Figure 3.**
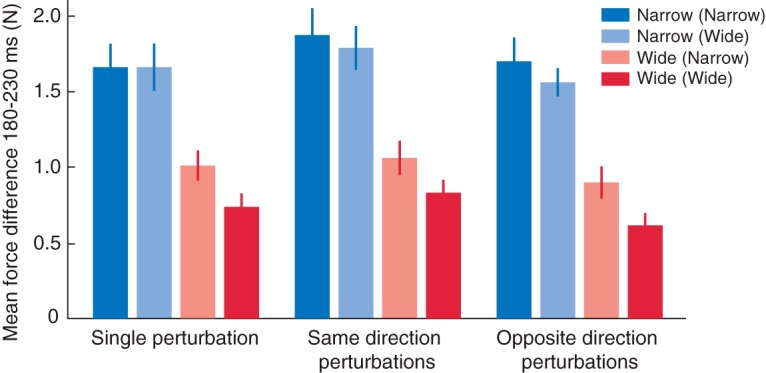
Visuomotor responses in experiment 2. Bars represent the mean force differences at a single hand averaged across the 180- to 230-ms interval following the cursor perturbation. Error bars represent ±1 SEM. Target sizes in parentheses indicate the size of the target of the other hand.

In our second study, we examined the extent to which visuomotor feedback gains are specified independently and in parallel for the two hands during bimanual reaching. To do this, we compared participants’ rapid corrective responses to lateral displacements of the cursor of one of the hands to the responses elicited by simultaneous shifts of the cursors of both hands. Bimanual reaches were performed to two-target configurations containing two narrow targets, two wide targets, or one narrow and one wide target. To eliminate any gaze-related effects (examined in experiment 1) and provide the cleanest test of the simultaneity of feedback gain specification for the two hands, we had participants fixate on a centrally located dot positioned in between the two targets (see Fig. 1*F*). Fig. 3 shows the mean corrective force difference in each experimental condition (computed using the same method as for experiment 1). To test for effects of target size, size of the target of the other hand, and perturbation condition, the force differences were subjected to repeated-measures ANOVA. As in the first experiment, we found that corrections were stronger during reaches toward narrow than toward wide targets (*F*_(1,13)_ = 99.9, *p* < 0.001). In addition, we observed that corrective forces at one hand showed interference of the width of the target of the other hand (*F*_(1,13)_ = 10.4, *p =* 0.007), with larger corrective forces when the other target was narrow than when the other target was wide. Notably, the influence of the width of the other target was more pronounced when the reach was performed toward a wide target (*F*_(1,13)_ = 19.6, *p =* 0.001). Planned comparisons revealed that corrective forces during reaching toward a narrow target did not differ between trials in which the other target was narrow and trials in which the other target was wide (*p =* 0.211, compare dark and light blue bars in Fig. 3), whereas corrective forces during reaching toward a wide target were larger when the other target was narrow than when the other target was wide (*p* < 0.001, compare dark and light red bars in Fig. 3). Although perturbation condition had no effect on the timing of the corrections (mean ± SEM *t*-test method, 167 ± 1 ms, *F*_(2,26)_ = 0.8, *p =* 0.441; extrapolation method, 140 ± 2 ms, *F*_(2,26)_ = 0.2, *p =* 0.856), it did influence the strength of the corrective forces (*F*_(2,26)_ = 9.7, *p =* 0.001). Although there was no significant difference in visuomotor feedback gain between trials with a single cursor perturbation and trials with perturbation of the two cursors in opposite directions (pairwise comparison *p =* 0.113), the gain was significantly greater when the two cursors were simultaneously shifted in the same direction than in single perturbation trials (pairwise comparison *p =* 0.001) or double perturbation trials with shifts in opposite directions (pairwise comparison *p =* 0.002). This pattern of effects is consistent with previous work on target displacements (Diedrichsen et al. 2004). The increased corrective responses observed when the two cursors were perturbed in the same direction may result from the overall stronger visual cue (e.g., consistent visual motion) compared with the single and opposite perturbation conditions.

In contrast to the results of experiment 1, we found no significant crosstalk between hands revealed by the force differences at the nonperturbed hand in single perturbation conditions (mean ± SEM 0.01 ± 0.02 N, all *p* > 0.05). The fact that crosstalk was observed in experiment 1 but not experiment 2 may reflect differences in gaze position. Central fixation (experiment 2) may allow for more independent processing of cursor motion because the left- and right-hand cursors are clearly in different hemifields (Alvarez and Cavanagh, 2005), whereas the cursor motion for one hand is close to midline when fixating on one of the two targets (experiment 1). Alternatively, participants may have more effectively suppressed crosstalk in experiment 2 because simultaneous cursor displacements could occur in opposing directions, in which case crosstalk would be particularly detrimental to goal attainment.

In summary, consistent with experiment 1, we found that visuomotor corrections to lateral shifts in cursor position were larger during reaches to narrow versus wide targets. Interestingly, however, the corrective response during reaches to wide targets was enhanced when the target of the other hand was narrow. We also found that, although there was no difference in the timing of the corrections, the forces at a single hand were larger in response to a shift of both cursors in the same direction compared with a single cursor shift, or a shift of both cursors in opposite directions. We discuss these and other findings below.

## Discussion

Goal-directed reaching movements are supported by several automatic reflexes that enable the motor system to rapidly respond to errors in target and hand position that may be sensed visually (e.g., Goodale et al. 1986; Saunders and Knill, 2003), proprioceptively (Scott, 2012), or even cutaneously (Pruszynski et al. 2016). Here we focused on visually detected errors in hand position and examined whether the visuomotor system exhibits a limited processing capacity across visual space. In our first experiment, we compared rapid visuomotor responses to a perturbation in the viewed hand position during bimanual versus unimanual reaching. We found that corrections were only 13% weaker during bimanual compared with unimanual reaching, whereas the sensitivity to target width and the timing of the corrections was not affected by the use of one or two hands. This suggests that visual-spatial processing at the two hands occurs largely in parallel. We further showed that visuomotor corrections were both faster and larger for perturbations of the cursor moving toward the fixated versus nonfixated target, highlighting the importance of the allocation of gaze during goal-directed reaching. In our second experiment, we examined whether the visuomotor system simultaneously specifies independent feedback gains for the two arms during bimanual reaching. Independent controllers have previously been suggested for the left and right arm during bimanual reaching (Diedrichsen et al. 2004) as well as for the index and thumb during grasping (Smeets and Brenner, 1999, 2001), but independent scaling of feedback gains has not been tested directly. We found that the responses at each hand to simultaneous perturbations of both hand cursors were independently adjusted to their corresponding target sizes, but also showed some interference of the size of the target of the other hand. This suggests that the specification of feedback gains for the two arms during bimanual reaching is largely, but not entirely, independent.

### Effect of Gaze Location on Visuomotor Feedback Gains

During unimanual reaching tasks, people naturally direct their gaze to the target during movement (Land and Furneaux, 1997; Johansson et al. 2001; Bowman et al. 2009). During bimanual reaches, however, gaze can be directed to only one location at a time. In the current study, we controlled gaze location and found that for both unimanual and bimanual reaches the corrective force response was greater, and implemented sooner, when perturbations occurred on the hand directed to the fixated, as opposed to the nonfixated, target. We can think of three possible explanations for this effect of fixation location. First, the visuomotor system might be better at detecting errors when the hand is moving toward the foveated location (Paillard, 1982). Beyond the magnocellular pathway being important for encoding dynamics, it has been shown that parietal neurons in area 7a respond best to visual stimulus motion toward the gaze location, regardless of stimulus position in the receptive field (Motter and Mountcastle, 1981; Steinmetz et al. 1987). Although these neuronal responses are consistent with the area playing a prominent role in processing visual optic flow patterns, they may additionally provide information about the direction of motion relative to the line of gaze, and thereby also support rapid visuomotor corrections (Steinmetz et al. 1987; Paillard, 1996). Second, foveating a target provides extraretinal (i.e., proprioceptive) cues about its position (Paillard, 1982; Desmurget et al. 1998), which presumably increases the certainty of the spatial representation of the foveated, compared with nonfoveated, target (van Beers et al. 1999). Previous studies have shown that the gain of motor corrections to target displacements increases with target certainty (Izawa and Shadmehr, 2008) and it seems plausible that higher spatial certainty of the target location would similarly increase the gain of corrections for cursor displacements. A third possibility is that the distance (i.e., visual angle) between the perturbation and the gaze position affects the magnitude and timing of the corrective response. It has been well documented that visual acuity decreases in an approximate monotonic fashion with increasing retinal eccentricity (Frisén and Glansholm, 1975) and, in our experimental setup, cursor perturbations occurred slightly further in peripheral vision when the perturbed hand was reaching toward the nonfixated versus fixated target. Although additional research will be required to test among these possibilities, it is notable that gaze position exhibits such robust influence over the automatic corrective response to cursor perturbations considering that, unlike target displacements, these perturbations (1) always occurred in peripheral vision, and (2) have been shown to be unaffected by the focus of attention (Reichenbach et al. 2014).

### The Resource Capacity of Visuomotor Feedback Gains during Reaching

The main goal of experiment 1 was to test whether there is a cost to visuomotor corrections during bimanual compared with unimanual reaching. Whereas the results of experiment 1 revealed a significant but small advantage for unimanual over bimanual reaching in terms of the magnitude of the visuomotor response, we also found that the timing of the response and its sensitivity to target width were not affected by whether one or two hands were moving. When examining the corrective responses to target displacements, Diedrichsen and colleagues (2004), using kinematic measures to assess corrective responses, found similar performance for unimanual and bimanual reaching in terms of the onset and size of corrections. Our observation of higher visuomotor gains during unimanual versus bimanual reaching, which was not found by Diedrichsen et al. (2004), might reflect differences in the evolution of feedback gains for errors in target versus cursor position (Reichenbach et al. 2014; Franklin et al. 2016). Alternatively, it may be that our use of force channel trials enabled us to detect subtle differences in gain that could not be detected using kinematic measures, which are contaminated by limb dynamics (Scheidt et al. 2000; Franklin and Wolpert, 2008). Overall, our results suggest that visuospatial processing at the two hands occurs largely, but not entirely, in parallel.

### Independence and Interaction of Feedback Gains during Bimanual Reaching

Consistent with previous research (Knill et al. 2011; Gallivan et al. 2016), corrective forces were greater during reaching to narrow than wide targets. To probe the extent to which the brain can independently specify, in parallel, different feedback gains for each hand, experiment 2 included conditions in which the two targets had incongruent sizes. Because the size of the target for the other hand is irrelevant for successfully completing a reach with one hand, the theory of optimal feedback control predicts that the responses at the two hands should be independent (Diedrichsen, 2007). Consistent with this prediction, we indeed found that the responses to a perturbation during reaching toward a narrow target did not depend on the width of the other target. In contrast, however, we found that the responses during reaching toward a wide target were enhanced when the other target was narrow compared with wide. A possible explanation for this asymmetry is that there is a tendency for the control policies of the two hands to interact but that this interaction can be suppressed when the primary task goal (i.e., hitting the target) is otherwise threatened. Thus, whereas the gain associated with the wide target may slightly increase when the other target is narrow, the motor system suppresses any tendency to decrease in the gain associated with the narrow target, when the other target is wide, because this would increase the probability of missing the target. This putative interaction between feedback gains may arise due to the multiplexing of ipsilateral and contralateral hand representations in the motor system (Donchin et al. 1998; Cisek et al. 2003; Hoshi and Tanji, 2004; Chang et al. 2008), which under many everyday circumstances supports bimanual control.

## Summary

Here, we show that visuomotor feedback gains for errors in hand position can be largely specified in parallel during bimanual reaching. However, our observation of a small, but significant, reduction in the reflex gains during bimanual compared with unimanual reaching suggests limitations in the resource capacity for processing visuomotor errors related to hand position across space. We found that the gain of the corrective response at each hand was strongly adjusted to the width of its own spatial goal. Although a small interaction between the gains at the two hands was observed under some conditions, we found that the motor system can specify these feedback gains completely independently when such control is required to satisfy task goals. Finally, we show that visuomotor feedback gains are strongly modulated by gaze position.

## Synthesis

Reviewing Editor: Li Li, The University of Hong Kong Decisions are customarily a result of the Reviewing Editor and the peer reviewers coming together and discussing their recommendations until a consensus is reached. When revisions are invited, a fact-based synthesis statement explaining their decision and outlining what is needed to prepare a revision will be listed below. The following reviewer(s) agreed to reveal their identity: He Cui, Michael Landy.

Both reviewers have consistent positive comments of the ms, e.g., while the research question is somewhat interesting, the research hypothesis is clearly supported by the data presented. Reviewer 1 wants some clarifications before the ms can be considered for publication. Both reviewers also have some specific comments that should be addressed in the revision. Below I attach the comments from the two reviewers.

Reviewer 1:

In the present study, the authors compared the visuomotor behaviors during unimanual and bimanual reaches. Based on the differential effects of perturbation on two tasks, the authors concluded that visuomotor corrective mechanisms largely operate in parallel across two limbs.

While the paradigm overall is cleverly designed (i.e. channel trials were introduced to eliminate effect of limb dynamics), it would be better to elaborate their finding with in-depth analysis to fully justify their conclusion.

General comments:

1. As mentioned, they observed different visuomotor feedbacks for channel and non-channel trials, please elaborate.

2. The perturbation distance is fixed (3 cm) in each trial, so the horizontal distance between hand position after perturbation and after reaching the target could be different due to target width. Since the visuomotor integration also relies on the end-point position, it's hard to conceive whether the target width or distance cause the significant difference.

Specific comments:

1. Fig. 2C and Fig. 3, it would be better to mark the significance in histograms.

Reviewer 2:

This is a modest but very clear paper that asks whether online control of a reach is independent between the two arms. The answer seems to be “mostly yes”, possibly depending on the error feedback being in separate visual fields (left and right). My comments are mostly minor. The conclusion is well-justified by the data. I describe the paper as “modest” because, well, it is just that. One simple question with a relatively simple answer. Two closely related experiments and appropriate statistical analysis. No modeling or other fancy analysis. An easy read.

Specifics:

* Line 44 or so: It's a little off-topic, but the model by Brenner and Smeets that suggests that precision grasping involves independent motor plans for thumb and index finger is an example of parallel motor planning and control.

* 119-120: Is this channel the same in terms of stiffness/damping as the channel described in the next paragraph? Why does it only control 7.5 cm rather than the 12.5 cm that takes the reach to the end of the occlusion, since it's supposed to control the position at emergence from the occluder?

* 135: Since the cursor abruptly shifts while it's visible at the end of the reach, doesn't this make the presence of a perturbation rather obvious? Might this affect the results (apparently not)?

* 164-166: You are citing Alvarez/Cavanagh, which is about a purely perceptual task (multiple-object tracking) as justifying a conclusion that this is sensorimotor. Well, MOT is a different task than multiple detections of errors, so this seems like weak logic.

* 179: “(i.e., 8 repetitions)”: Well, it's 16 repetitions of the unperturbed reaches.

* 197: Were there no misses at all on non-channel trials?

* 211: “a value GREATER than zero”: Do you mean a signed value greater than zero or whether the absolute value of the force was significantly non-zero? I'm guessing that previous literature indicates that cross-talk always has the same direction, but I don't know that literature...

* Half of the trials were not channel trials, and yet this half of the data is never discussed, analyzed, nor are those trajectories plotted. Why?

* 282: I'm a bit unconvinced by the analysis of the time of correction. That analysis depends on the significance of a deviation that, in turn, depends on the size of the eventual correction (i.e., almost by definition larger corrections will become significant earlier)
